# Hepatic encephalopathy as an indication or contraindication to liver transplant?

**DOI:** 10.1007/s11011-025-01614-w

**Published:** 2025-04-15

**Authors:** Mette Munk Lauridsen, Jasmohan S. Bajaj

**Affiliations:** 1 Division of Gastroenterology, Hepatology and Nutrition, Virginia Commonwealth University and Richmond VA Medical Center, Richmond, VA USA; 2 Department of Gastroenterology, University Hospital of South Denmark, Finsensgade 35, Esbjerg, 6700 Denmark

**Keywords:** Cognitive reserve, Gut-brain axis, Calcineurin inhibitors, Multi-modal MRI, Three villages, Alcohol

## Abstract

Hepatic encephalopathy (HE) presents a significant challenge in liver transplantation (LT). On the one hand, LT can provide a curative treatment for HE by addressing its underlying cause, suggesting HE should be a strong indication for LT. Conversely, the severity of HE may reflect advanced liver disease and significant neurocognitive impairment, potentially complicating post-transplant outcomes and raising concerns about its suitability as an indication. This review will provide helpful insight to the hepatologist deciding whether HE should be considered an indication or a contraindication to liver transplantation in their patient. It gives an overview of the burden of HE pretransplant, HE’s current status in the transplant listing process, and pre- and post-transplant cognitive issues to be mindful of. The main take-away messages are that pre-transplant HE should be managed aggressively, that neurodegenerative disorders and other differential diagnoses to HE should be thoroughly excluded, and that immunosuppressants can cause new onset cognitive issues post-transplant and should be monitored closely. In the future, objective measures of HE severity should be included in the MELD score to enhance the fairness and efficacy of transplant listings, ensuring those with cirrhosis complicated by HE receive timely and appropriate treatment.

## Introduction

Hepatic encephalopathy (HE) represents a complex neuropsychiatric syndrome associated with liver dysfunction, characterized by cognitive, psychiatric, and motor impairments (Häussinger et al. 2022). The pathophysiology of HE is multifactorial, involving ammonia dysmetabolism, systemic inflammation, and alterations in neurotransmission, leading to significant morbidity and mortality among patients with liver disease (Häussinger et al. 2022). Liver transplantation (LT) remains the definitive treatment for end-stage liver disease (ESLD), offering a potential cure and significant improvement in quality of life. However, the presence of HE poses unique challenges in the context of LT, raising the question of whether HE should be considered an indication or a contraindication to transplantation. On the one hand, LT can provide a curative treatment for HE by addressing its underlying cause, suggesting HE should be a strong indication for LT(Ahluwalia et al. 2016). Conversely, the severity of HE may reflect advanced liver disease and significant neurocognitive impairment, potentially complicating post-transplant outcomes and raising concerns about its suitability as an indication. This review will provide helpful insight for the hepatologist deciding whether HE should be considered an indication or a contraindication to liver transplantation in their patient. We will provide a concise overview of the burden of HE pre-transplant, its current role in the transplant listing process, and cognitive issues both before and after transplantation. The primary focus will be on Type C HE, the most common type in patients with chronic liver disease. This review is partly based on the presentation by JSB at the 2023 ISHEN meeting in Germany.

### The multi-level burden of hepatic encephalopathy

**Fig. 1:**
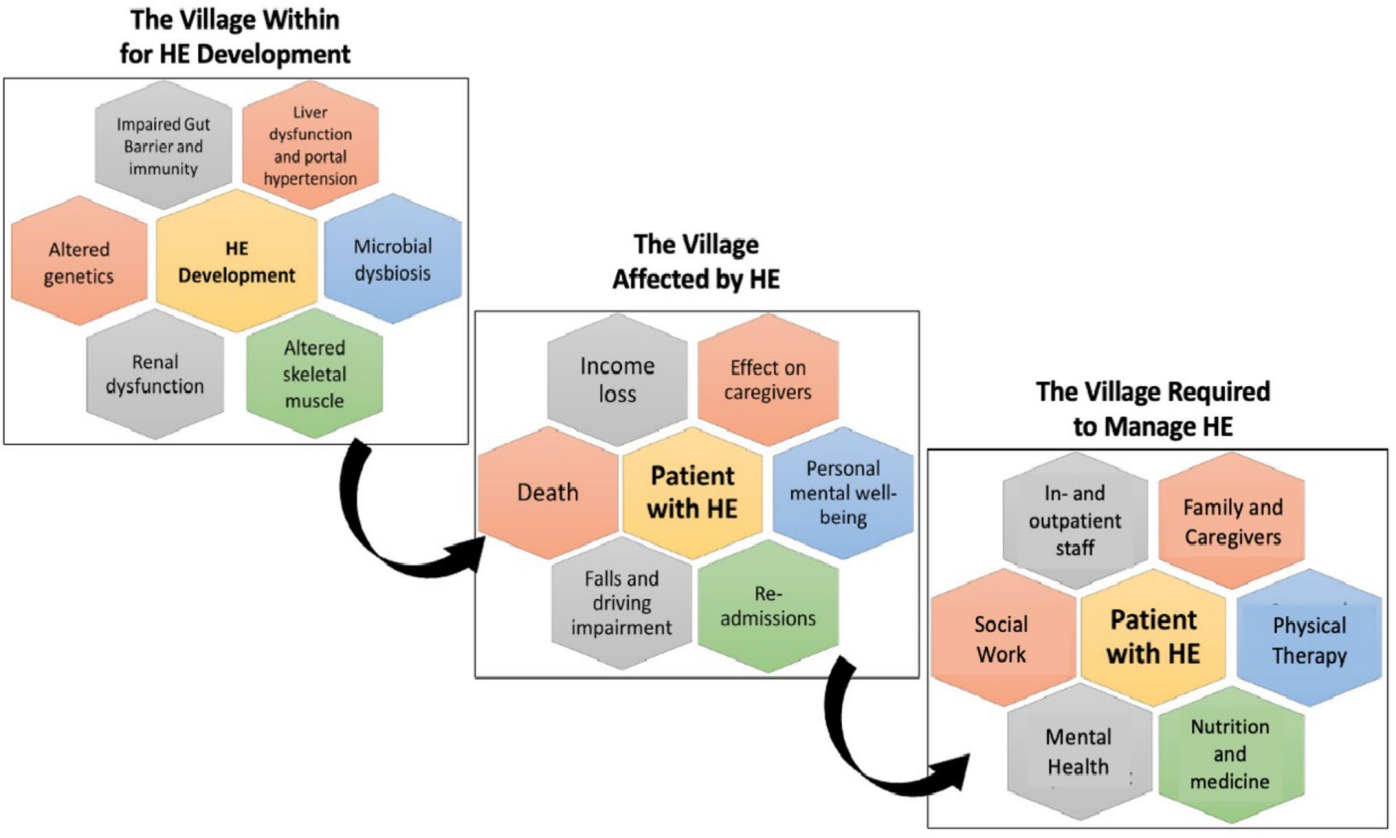
‘The three villages of HE’ represent the cross-cutting burden of HE: The complex pathogenesis (top hexagons), the multi-level impact on the patient’s life (middle), and the need for a multidisciplinary management effort (bottom)

HE significantly disrupts the lives of patients and their caregivers and requires a multidisciplinary management effort (Fig. 1) (Fabrellas et al. 2020; Montagnese and Bajaj 2019; Ridola et al. 2018; Bajaj et al. 2011; Bajaj 2021). The impact of HE on families and caregivers is crucial when assessing the disease’s overall burden and the patient’s suitability for LT. Cognitive decline, a hallmark of HE, hampers the ability of patients to engage fully in activities of daily life, resulting in both medical challenges and psychosocial stress for patients and their support networks. This loss of cognitive function and diminishing independence and socioeconomic status reduce the patient’s sense of self-efficacy (Ladegaard Grønkjær et al. 2018). Furthermore, HE has been observed to adversely affect employment and the financial condition of patients, while caregivers experience feelings of being trapped and concerns for their health. HE is further correlated with hospital admissions, sarcopenia, and falls (Yildirim 2017; Ha et al. 2022). Consequently, families frequently bear the weight of the patient’s deteriorating autonomy and functionality (Sørensen et al. 2024). Therefore, the social determinants of health play a pivotal role in HE and cirrhosis, shaping the family’s capacity to manage these significant stressors while preserving their own health. The deleterious effects of HE raise the question of whether patients with recurrent episodes should receive higher priority for transplant listing. Addressing the complex challenges associated with HE necessitates a comprehensive, multidisciplinary approach. Such care teams should ideally include outpatient medical staff (comprising GI/Hepatology physicians, nurses, and advanced practice providers), inpatient teams (including a dedicated hospital medicine team), and specialists in nutrition, social work, mental health, and physical therapy (Fig. 1) (Bajaj 2021). While such a coordinated care strategy is established practice for hepatocellular carcinoma, it has not been formally adopted for HE, underscoring the need for a standardized approach in the management of this condition.

### Hepatic encephalopathy is not systematically considered at transplant listing

The allocation of organs for transplantation is currently guided by the Model for End-Stage Liver Disease (MELD) score. This system prioritizes patients based on the severity of their liver disease but does not explicitly incorporate the presence or severity of HE (Acharya and Bajaj 2021). The MELD score’s lack of specificity for HE is problematic because, as previously mentioned, HE significantly affects patients’ quality of life and has implications for transplant urgency and post-transplant outcomes. The absence of a systematic method for incorporating HE into the MELD score stems from a lack of consensus on how to objectively quantify HE and the absence of standardized criteria for its inclusion in the allocation process. A potential solution to this problem is using hospitalization records to document HE episodes. This would allow for a pragmatic assessment of the disease’s burden by identifying patients with grade 2 or higher HE episodes. By providing additional MELD points to patients with documented recurrent HE who meet specific criteria—such as consistent medication adherence, management of precipitating factors, and the absence of treatable portosystemic shunts—there is an opportunity to refine the organ allocation process. The availability of a specific International Classification of Diseases (ICD) code and the systematic documentation of HE episodes according to grade, type, time course, and precipitating factors, as suggested by guidelines, would aid this process (Häussinger et al. 2022; Vilstrup et al. 2014). This proposed modification to the MELD aims to enhance fairness in organ allocation, ensuring that patients with HE have equitable access to transplantation and the opportunity for definitive treatment. The proposed changes would necessitate alterations in national transplantation policies and the development of new guidelines to accurately reflect the severity of HE in the MELD score. However, a recent study showed that even this does not fully solve the problem of HE not getting listing priority (Silvey et al. 2025) A drawback to such an approach is the potential for inflation of HE severity, as the subjective nature of assessment could lead to unfair advantages in the organ allocation process. To mitigate this, objective HE measures, such as specific biomarker panels in combination with neuropsychiatric assessments, could be incorporated into the pre-transplant workup. Therefore, research aiming to provide such objective HE measures is welcomed. Balancing the need for objective assessment with the complexity of HE’s presentation is crucial for evolving the organ allocation system to better serve patients with end-stage liver disease complicated by HE.

### Pre-transplant cognitive problems — it is not always HE

**Fig. 2:**
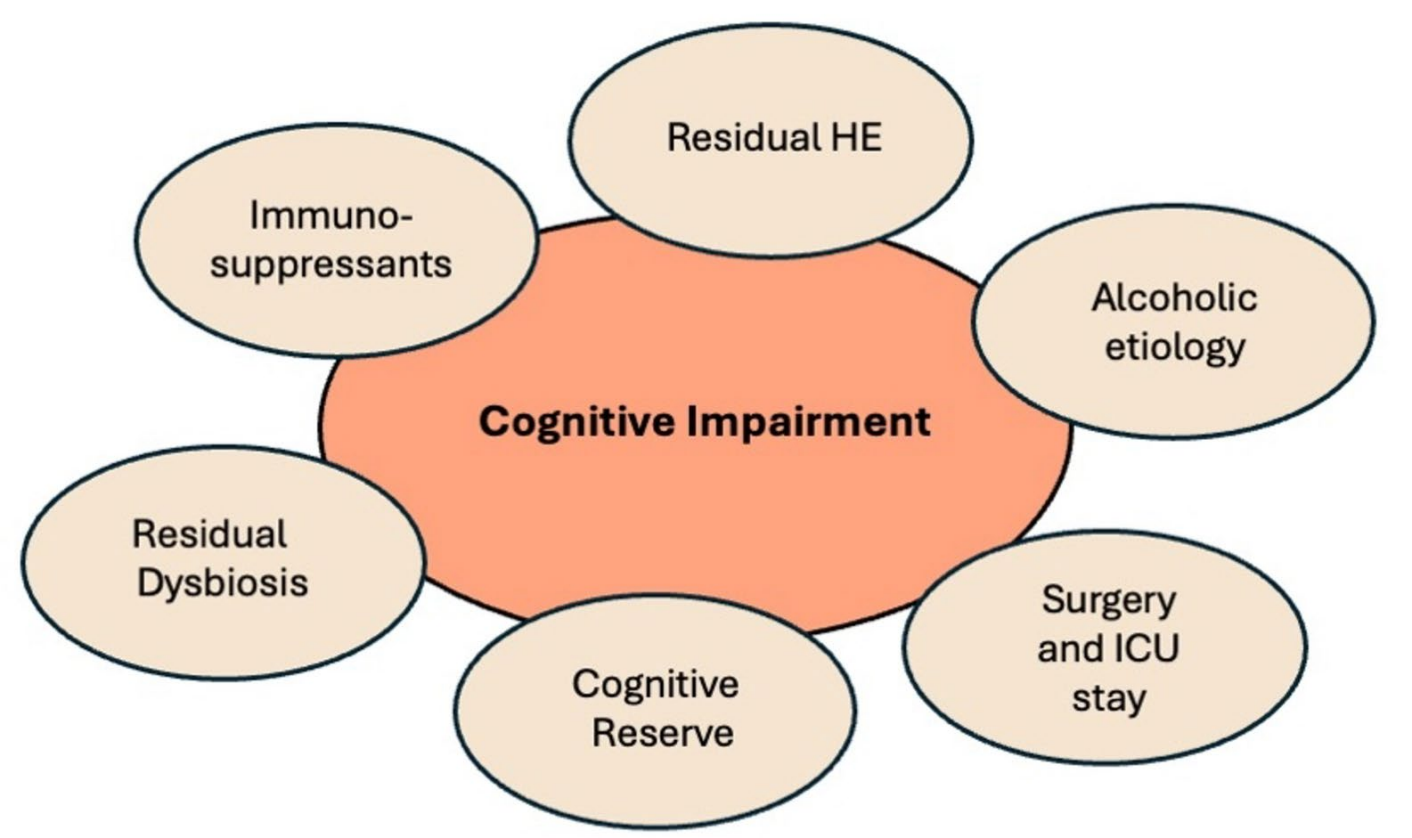
Important factors involved in cognition after liver transplantation

Cognitive impairment in cirrhotic patients is frequently attributed to HE, leading to a common clinical practice where lactulose is prescribed reflexively, often without specialized diagnostic testing. This assumption may overlook other potential causes of cognitive decline, which is particularly concerning given the aging cirrhotic population and the prevalence of comorbid conditions (Fig. 2). Reflexive treatment of presumed HE without a comprehensive evaluation can result in mismanagement and a failure to address the true etiology of the cognitive deficits (Bajaj et al. 2024). Efforts to improve diagnostic accuracy, such as establishing dedicated HE clinics, provide an avenue for detailed neuropsychological assessments (Nadeem et al. 2023). These clinics offer critical insights by conducting thorough reviews of psychoactive medications, evaluating comorbidities, and confirming medication adherence. However, the implementation of such clinics is often hindered by challenges related to logistics, funding, personnel training, and the need for procedural standardization. Still, for other cirrhosis-related problems, such as oesophageal varices, standardized, resource-heavy screening procedures are in place in most clinics and have changed the disease course. Given the multi-level impact of HE, it deserves the same level of attention.

A thorough diagnostic process is needed because neurocognitive dysfunction in cirrhotic patients being evaluated for liver transplantation can stem from various risk factors. Patients with alcohol-related liver disease, for instance, have been shown to experience more significant neurocognitive alterations and detectable cortical lesions following trans-jugular intrahepatic portosystemic shunt (TIPS) procedures, as seen on brain MRI scans, compared to those with non-alcohol-related liver disease (Ahluwalia et al. 2015). Moreover, extensive epidemiological studies suggest a link between hepatitis C infection and a heightened risk for neurodegenerative diseases such as Parkinson’s and Alzheimer’s Disease (Yaow et al. 2023; Narváez-Bandera et al. 2024; Bruno et al. 2023). A critical factor in a patient’s ability to cope with these neurocognitive challenges is their cognitive reserve, which is influenced by factors such as educational attainment, psychometric intelligence, engagement in leisure activities, and overall lifestyle. Notably, cirrhotic patients who have a high cognitive reserve tend to maintain a better quality of life, even in the face of advanced liver disease (Amodio et al. 2017; Patel et al. 2015; Schiff et al. 2014).

### Post-transplant cognitive problems

Cognitive rehabilitation post-LT is a contentious topic, given that up to 30% of post-LT patients experience neurological symptoms (Albhaisi and Bajaj 2020; Khalil et al. 2020; Saner et al. 2007). This incidence is substantially higher compared to the postoperative phase of cardiac and renal transplants, where the rates are 4% and 0.5%, respectively. While these cognitive issues were historically ascribed to HE sequelae, it is now recognized that a broader spectrum of factors plays a role.

In a prospective, observational study, 50 patients on the waiting list for LT were examined in an outpatient setting before LT and 6 and 12 months after LT with the PHES. It was found that cognitive dysfunction developing after LT was independent of a patient’s HE status before LT, suggesting that cognitive disturbances in LT patients are not always residual symptoms but new onset. (Tryc et al. 2014). As the post-transplant timeline progresses, the etiological factors for neurological complications evolve, encompassing risks like surgical reinterventions, the necessity of postoperative renal replacement therapy, extended periods of mechanical ventilation, and severe post-operative infections (Weiss and Thabut 2019). The long-term cognitive outcomes after LT are heterogeneous—some patients experience sustained cognitive improvements, while others face persistent or progressive decline. It can be difficult to delineate between residual HE versus new post-transplant encephalopathy, and we here discuss some of the factors pertinent to cognition post-LT (Fig. 2).

#### Surgery and ICU stay

The initial post-operative period can pose acute neurological challenges, including seizures, strokes, infections of the central nervous system, and weaknesses acquired during intensive care unit (ICU) stays. These complications are among the most frequently encountered short-term neurological issues, significantly impacting the immediate post-LT recovery phase. They may, in part, be attributable to the substantial alterations in cerebral blood flow and systemic hemodynamics that occur during major surgery like LT and the effects of anesthesia (Weiss and Thabut 2019). In the early phase after transplant, but also later, systemic inflammation is hypothesized to play a role in postoperative cognitive dysfunction or delirium.

#### Alcohol-related etiology

As stated earlier, brain reserve is affected by many variables and is a strong determinant in a patient’s ability to maintain cognitive function during and after LT. Chronic alcohol overuse has a strong negative effect on brain reserve, and it has been shown that this caused increased cortical damage and brain edema and scores worse on cognitive tests than patients with non-alcohol etiology after elective TIPS placement (Ahluwalia et al. 2015). Alcohol’s effects on brain reserve are manifested as grey matter atrophy and microstructural damage in the white matter. It has been shown that even with continued abstinence, some neuronal loss may be irreversible, and Diffusion Tensor Imaging studies show that white matter reduction is seen in relapse of alcohol overuse (Zahr and Pfefferbaum 2017). It is discouraging that abstinence from alcohol in the setting of cirrhosis promotes limited improvement in brain function. This reflects that the patients who develop alcohol-related cirrhosis typically have a sustained pattern of drinking over several decades, which causes significant organic brain damage and reduces the brain’s ability to recover from toxic exposure over time (Ahluwalia et al. 2015).

#### Immunosuppressants

The neurotoxicity of immunosuppressant drugs, particularly at high levels, is another well-documented source of post-transplant cognitive confusion. The more subtle neurocognitive impairments that occur farther out from transplant are often attributed to calcineurin inhibitors, though they cannot account for all the reported abnormalities (Dirks et al. 2020, 2021). Another cause of new-onset cognitive symptoms attributable to immunosuppressants is neuroinfections. In the first several months after LT, other causes of neurological dysfunction tend to predominate, including infections from organisms such as the JC virus, aspergillus, Nocardia, mycobacterium tuberculosis, and cryptococcus neoformans (Weiss and Thabut 2019).

#### Dysbiosis

The gut microbial composition has also been associated with cognitive dysfunction before and after LT. In a single-center study that enrolled 45 patients who had undergone LT, a significantly improved health-related quality of life (HRQOL), psychometric hepatic encephalopathy score (PHES), and increased microbial diversity were seen in patients after LT compared with baseline. However, there was continued dysbiosis and HRQOL/cognitive impairment after LT compared with controls in 29% who did not improve their PHES after LT. Patients with post-transplant cognitive impairment were found to have significantly higher relative abundance in Proteobacteria and a decrease in Firmicutes post-LT. At the same time, the reverse occurred in the group that improved (Bajaj et al. 2017). The study concluded that LT improves gut microbiota diversity compared with pre-LT baseline, but residual dysbiosis remains compared with controls. Another study found that LT improved gut microbial functionality through favorable changes in bile acids, ammonia, and endotoxins. The study enrolled 40 patients with cirrhosis on the LT list and followed them until six months post-LT while performing both cognitive tests and microbiota composition tests at both visits. The study showed a significant improvement in cognition with increased microbial diversity, decreased potentially pathogenic toxins, and reduced endotoxins post-LT. There was evidence of greater bacterial action (as seen by higher secondary bile acids) post-LT and reduced serum ammonia, which corresponded to improvements in cognitive function (Bajaj et al. 2018). Additionally, increases in urinary phenylacetylglutamine (PAG) post-LT also correlated with cognitive improvement. Studies on LT and its effect on the microbiota have also shown effects on physiologic systems other than the nervous system. For instance, one study found that the generation of trimethylamine-N-oxide (TMAO), which is abundant in red meat and can accelerate atherosclerosis, is increased six months post-LT. In patients with cirrhosis, cognitive dysfunction is linked with TMAO levels. Improved liver function likely leads to an increase in the relative abundance of the family Enterobacteriaceae, which converts dietary carnitine to TMAO. It is also likely that the rise in TMAO in patients at the early post-LT stage represents a manifestation of a return to normalcy of the liver and that post-LT cardiovascular disease is unlikely to occur within several months after transplant. Therefore, longer follow-up is required to determine whether this TMAO increase persists and is related to atherogenic events later in the post-LT course (Bajaj et al. 2018).

#### Residual HE

Low-grade brain edema, cognition, and quality of life generally improve after LT. A study using MRI and magnetization transfer ratios, a measure of brain edema, reported progressive amelioration from pre-LT to one-year post-LT (Ahluwalia et al. 2018). This was backed up by a single-center study of sixty-six patients that showed a significant improvement from pre-LT baseline in most patients from a neuropsychological, functional, and multi-modal MR perspective. Functional MRI showed that patients required less effort to perform the same actions post-LT. In addition, a reversal of pre-transplant ammonia-associated changes was seen on MR spectroscopy, and white matter integrity improved on MR diffusion tensor imaging after LT. However, the study also showed that while patients with cognitive impairment improved significantly after transplant, they remained more impaired compared to those who did not have pre-transplant cognitive impairment (Ahluwalia et al. 2016). Acknowledgment has grown that, despite the acute manifestations of HE being largely reversible, residual metabolic disturbances can lead to longer cognitive recovery time or even permanent neurological harm. This observation was backed up by a longitudinal study involving 34 patients, using the Repeatable Battery for the Assessment of Neuropsychological Status (RBANS) before and after LT. The study found that individuals with a pre-LT history of HE could regain normal cognitive functions and an enhanced quality of life within five years post-transplant (Hopp et al. 2019) while patients with no prior HE showed recovery already after one year. Moreover, the presence of “dementia-like” alterations in individuals with severe HE prior to LT supports the notion of a lasting impact of HE on cognitive functions, affecting areas such as working memory, response inhibition, and learning capabilities, as well as a likely overlap (Adejumo et al. 2023). This evidence underscores the intricate interplay between HE's lasting cerebral effects and the potential for cognitive restoration following LT. This knowledge must be considered when counseling patients and their caregivers about the potential for cognitive recovery after transplant.

### Workup and management strategies post-transplant

Presently, there are no recommendations from professional or learned medical societies for preventing neurological or neurocognitive complications after LT. Measures that have been proposed to avoid these complications include the following:

- treating pre-LT HE aggressively through medical therapy or embolization of spontaneous portosystemic shunts,
- slowing the rise of sodium levels to reduce the risk of post-LT osmotic demyelination syndrome.
- ensuring dementing processes are excluded pre-LT.
- assessing/treating sarcopenia aggressively.

However, there is no data investigating the usefulness of such strategies (Bajaj et al. 2023). Modulating the systemic inflammation response in the pre-, peri-, and post-LT periods is a promising approach; however, none of the proposed strategies in the ICU literature have demonstrated any discernible benefit.

In patients where there is concern for another neurologic disorder, it may be useful to undergo a systematic neurological evaluation prior to LT to uncover undiagnosed neurocognitive impairment. Brain imaging, especially MRI with standard sequences, may demonstrate chronic vascular changes or brain atrophy in such patients. Neuropsychological testing could reveal a profile that is not typical of HE but one that is more suggestive of a vascular origin or neurodegenerative disorder. Biomarkers of neurodegenerative disorders that are used with increasing frequency in neurology could help diagnose a neurodegenerative disorder. While abnormal findings may increase the risk of prolonged neurologic impairment after LT, whether they should preclude access to LT remains unknown. Additionally, an overly exhaustive workup could delay access to LT with potentially deleterious consequences for the patient’s outcome. On the other hand, patients who have been referred for LT specifically for persistent HE may have been misdiagnosed with HE, and therefore, a pre-LT neurologic evaluation may be helpful to exclude such patients with previously undiagnosed cerebral dysfunction who have a high probability of decompensation after LT.

## Summary

Cognitive impairment and brain reserve, rather than simply prior HE, are major determinants of post-LT cognitive function and should be investigated and treated to optimize the pre-LT patient who is undergoing transplant evaluation. The reversibility of post-LT neurological changes depends on a multitude of pre-, peri-, and post-operative factors that are often difficult to influence. Regardless, it is critical to screen for and further investigate patients in whom potential underlying neurodegenerative disorders are suspected prior to LT because of the high risk of neurologic decompensation during the process of LT. Various biomarkers related to specialized brain imaging, microbial composition, and microbial function change pre- and post-LT have been shown to predict outcomes related to cognitive dysfunction. Other social factors, such as high education, good social support, and higher socio-economic status, are all associated with improved neurologic outcomes after LT and should be considered by clinicians when counselling patients. At present, the current organ allocation score (the MELD 3.0) does not include HE, which worsens mortality. However, further research is needed to operationalize how and if patients with HE should be given extra points to increase their odds of receiving a timely transplant. These patients are at high risk for decompensation and mortality in the short-term and long-term without LT, which necessitates the development of a framework that incorporates HE as a criterion for listing. Until then, it is essential to ensure that affected patients are adequately diagnosed, treated, and optimized in the pre-LT period to maximize their chances for positive long-term outcomes.

## Data Availability

No datasets were generated or analysed during the current study.
